# Breastfeeding in Tzeltal indigenous communities in Mexico: embodied and participatory experiences for policy recommendations

**DOI:** 10.1186/s13006-026-00815-y

**Published:** 2026-02-07

**Authors:** Ximena A. González-Grandón, Joao Gabriel Almeida, Sonia Hernández-Cordero, Mónica Ancira-Moreno, Marlene Peters-Castilla, Mariana Colmenares-Castaño

**Affiliations:** 1 Universidad Iberoamericana, Ciudad de México, Centre for Interdisciplinary Research for the Development of Education CINIDE, Prolongación Paseo de la Reforma 880, Colonia Lomas de Santa Fe, Álvaro Obregón, Ciudad de México, 01219 México; 2 Colegio de la Frontera Sur ECOSUR, Departamento de Agricultura, Sociedad y Ambiente, San Cristóbal de las Casas, México; 3 Universidad Iberoamericana, Ciudad de México, Research Center for Equitable Development EQUIDE, Prolongación Paseo de la Reforma 880, Colonia Lomas de Santa Fe, Álvaro Obregón, Ciudad de México, 01219 México; 4 Universidad Iberoamericana, Ciudad de México, Health Department, Prolongación Paseo de la Reforma 880, Colonia Lomas de Santa Fe, Álvaro Obregón, Ciudad de México, 01219 México; 5 Chelsea and Westminster Hospital, Cheyne Development Centre, London, UK; 6 Universidad Nacional Autónoma de México, Faculty of Medicine, Ciudad de México, México; 7 Instituto de Filosofía y Ciencias de la Complejidad, Santiago, Chile; 8 Universidad Iberoamericana, Observatorio Materno Infantil, Ciudad de México, México

**Keywords:** Tzeltal Indigenous community, Embodied practices, Communitarian care, Situated breastfeeding promotion, Altos de Chiapas, Territory, Maternal narratives

## Abstract

**Background:**

As a pluricultural country, Mexico must promote breastfeeding through intercultural approaches. Although existing research remains limited, the exclusion and precarious living conditions experienced by many Indigenous communities render such efforts both challenging and essential for reducing inequalities in breastfeeding support. Moreover, while low breastfeeding rates at both global and local levels have been extensively documented through quantitative studies, the lived experiences of Indigenous mothers remain highly variable and underexplored. To date, qualitative research centered on Indigenous breastfeeding mothers’ perspectives is scarce. This paper presents a case study of breastfeeding practices within a Tzeltal Indigenous community, examining the embodied, co-participatory, and situated dimensions of maternal care.

**Methods:**

Using qualitative methods and Interpretative Phenomenological Analysis within the ‘Operational Research Group and Participatory Inquiry´ framework. A total of 19 Tzeltal-speaking mothers who were breastfeeding their children participated in the study, conducted on November 12, 2024, in the Tzeltal community of San Juan Cancuc, Chiapas. Data were collected through group sessions documented with field notes and audio recordings. The material was analyzed using conventional content analysis with emergent coding, grounding interpretations in participants’ lived, embodied, affective, communal, and contextual breastfeeding experiences, allowing categories to be inductively generated from participants’ narratives.

**Results:**

The findings highlight that breastfeeding practices within a Tzeltal Indigenous community are not merely an individual act but a deeply relational, embodied and communitarian experience shaped by sociocultural, cosmogonic and spatial-contextual factors.

**Conclusions:**

This qualitative study, grounded in an intercultural perspective and centered on Indigenous mothers’ breastfeeding experiences, advocates for a more nuanced, interculturally oriented approach to breastfeeding promotion, protection, and support -one that recognizes the affective, embodied, and collective dimensions of maternal care. By attending to local beliefs, cosmogonies, and shared practices surrounding traditional breastfeeding, the study offers crucial insights that contribute to advancing reproductive justice for women, infants, and their communities, while underscoring the importance of intercultural strategies in the design and implementation of breastfeeding policies in a pluricultural country such as Mexico.

## Background

Breastfeeding is a unique source of nourishment for babies, not only because of its nutritional value but also due to its immunological and emotional impact on mothers, babies, families, and the broader environment. As a practice involving intimate bodily interaction –skin-to-skin or mouth-to-skin contact between mother and child–breastfeeding extends beyond physical health to foster emotional bonding and strengthen caregiving relationships. Its well-documented benefits have led to strong international and national policy support for humane breastfeeding practices [1–3].

The global strategy for infant feeding recommends exclusive breastfeeding for the first six months of life followed by continued breastfeeding, alongside safe and nutritious complementary food up to two years of age and beyond if desired. In Mexico, the breastfeeding rates remain low, and current support interventions are inadequate across all socioeconomic levels. Only 33.6% of infants under six months are exclusively breastfed, with slightly higher rates in urban (35.4%) than rural areas (28.9%), while 45.1% of children aged 12 to 23 months continue breastfeeding [4], falling far short of the exclusive breastfeeding updated global goals by 2030 [2]. Despite some progress, more comprehensive policies and interventions are needed.

Mexico, being a multicultural country, must consider the promotion of breastfeeding in intercultural codes. Although we have data from research about minority and indigenous groups, it is still scarce [5–7]. The vast majority of the indigenous communities in Mexico live in precariousness conditions and exclusion. Conducting research in these communities represents a challenge and data collected is valuable. We should avoid epistemic extractivism –referring to the extraction and reinterpretation of Indigenous knowledge through Western frameworks, which often disregards context, removes political meaning, and reinforces dominant worldviews [8]. Instead, research efforts should aim to foster reciprocal and context-sensitive knowledge exchanges that are meaningful, respectful, and beneficial to all parties involved.

We approached the indigenous Tzeltal community in San Juan Cancuc, to listen to the voices of Tzeltal women who embody the breastfeeding experience, sharing it body-to-body with their infants and community members. We find it relevant to emphasize an embodied perspective on breastfeeding, as it centers the analysis on the feelings and doings that make breastfeeding possible.

This territory covers approximately 15,000 square kilometers and is home to around 450,000 people. The traditional Tzetzal territory is located northeast and southeast of the city of San Cristóbal and includes the municipalities of San Juan Cancuc, Chanal, Oxchuc, Tenejapa and Altamirano; to the north: Sitalá, Socoltenango, Yajalón, Chilón, Ocosingo, Amatenango del Valle and Aguacatenango (municipality of Villa de las Rosas). Despite facing high levels of poverty, marginalization, and limited access to basic services [9], the community maintains strong traditional governance through *usos y costumbres* (customes and traditions). Historically, it has been a center of Indigenous resistance, notably during the 1712 Tzeltal uprising, with ongoing efforts to preserve cultural, territorial, and linguistic autonomy. Given our interest in understanding breastfeeding as an embodied, social, and collective practice, this community is particularly relevant, for three reasons:

First, specially San Juan Cancuc, it is characterized by a profound indigenous identity, with approximately 99% of residents identifying as indigenous, primarily Tzeltal Maya [10]. Remarkably, nearly all residents (99.9%) speak the Tzeltal language, underscoring the region’s linguistic and cultural homogeneity [10]. So, they maintain distinct cultural dynamics, with language, ancestral beliefs, and cosmogonic worldviews remaining vibrant and deeply influential despite modern influences [11]. Among their intergenerationally transmitted customs and traditions, the role of the body and body-to-body interaction during breastfeeding and parenting is especially central and is organized in unique ways. As a means of embodied and traditional knowledge that is transmitted intergenerationally, lived and continuously observed within the community. Indeed, the senses, muscles, and shared affectivities are engaged differently compared to Western societies and urban contexts. For instance, touch and auditory communication play a prominent role, while mutual eye contact is less central within social interactions [12]. Also, Tzeltal infants and toddlers are carried by their caregivers, on their backs, using a *rebozo* (traditional Mexican shawl) allowing constant closeness. This proximity also enables them to nurse freely and on demand while interacting and engaging with the activities taking place around their caregiver.

Second, in Tzeltal communities, collective identity is deeply embedded in communitarian social structures. This is reflected in language, where individual ownership is downplayed in favor of shared belonging. For example, speakers say jk’altik (“our milpa”) rather than jk’al (“my milpa”), as using the collective form affirms shared responsibility and mutual care. Similarly, they refer to parents as jMejTatik (“our Mothers-Fathers”), extending the concept of kinship to the entire community [13]. This linguistic and symbolic practice reinforces a collective ‘we,’ where even individual labor, such as maize production, is framed as a communal contribution [13]. The same principle applies to breastfeeding, where a mother’s nurturing role is understood not just as personal care but as a vital act that sustains the collective. Through these embodied interactions, mothers reaffirm their connection to the community, emphasizing that nourishment –whether through milk or food– is a shared responsibility and a fundamental expression of collective identity [14].

And third, in Tzeltal indigenous communities, territory is a fundamental part of identity, not only for its ancestral and symbolic significance but also for its role in sustaining communal life [15]. This shared territory fosters open and confident breastfeeding, as communal spaces naturally support body-to-body interaction between mothers and infants. Unlike urban environments, where caregiving is often segregated from other activities, indigenous spaces encourage continuous mother-infant proximity.

The ultimate aim of this research is gaining a deeper understanding of their breastfeeding embodied and affective practices, form their own traditions, within their own territories, and in contrast with urban environments. Also, that allows us to develop interculturally relevant breastfeeding promotion strategies in a multicultural country like Mexico.

### Breastfeeding bodies, communities and spaces

We begin by clarifying what we mean by embodied experiences of breastfeeding. Our understanding assumes that bodies are anatomical, physiological, experiential, and culturally shaped entities, and that the specific features of each woman’s embodiment vary according to her particular social location [16]. Thus, embodiment in our approach, highlights the full body’s role within research [17]. On one hand, it highlights lived experience of Tzeltal women as the main starting point, the question of what it feels like mothering through bodily experiences that are infused with affectivity –broadly about feelings, emotions, and sensibility [18].

On the other, it considers breastfeeding as a practice, a daily repertoire of caregiving behaviors that Hausman [19] describes as an embodied form of knowledge. It is not an individual act but a dyadic activity that inherently involves at least two active bodies learning to share sensations and affects. Indeed, breastfeeding is an inherently embodied and relational experience, where the mother and infant connect through shared sensations, emotions and behaviors. The mother learns to hold her child chest-to-chest, and her body –nipple, skin, breast milk, and arms– becomes intertwined with the infant’s mouth for prolonged and repetitive periods. This physical connection mirrors the umbilical cord’s role in nourishing the fetus, as the flow of breast milk similarly sustains and links their bodies [20].

Recognizing embodiment in breastfeeding means acknowledging the contextual practice of a mother’s daily lived experience of affectivity and caregiving labor, with its continuous and demanding actions. It also involves understanding how breastfeeding interactions are shaped by social and cultural contexts, which requires considering cultural traditions as well as economic and political structures [16, 19].

Furthermore, the research that has begun to consider the category of embodiment calls for listening to the lived experiences of women from non-industrialized or rural communities. For instance, when discussing breastfeeding, women from different parts of the world emphasize the need for realistic, practical, and horizontal messaging over idealized or prescriptive approaches [21]. Similarly, Keith et al. [22] report narratives from women highlighting the value they place on educational strategies that acknowledge their breastfeeding bodies—for example, those that address experiences of warmth and pleasant emotions, or sensations of nipple pain and feelings of rejection while breastfeeding. Likewise, Eni et al. [23] emphasize the importance of focusing on the body-to-body relationship between mother and infant within cultural contexts with specific traditions. Much of this research highlights a gap in public health campaigns, which often overlook these embodied and contextual realities.

For us, recognizing embodiment implies these described dimensions, and drawing on Merleau-Ponty’s phenomenology, to emphasize the explicit body-to-body coupling involved in breastmilk sharing –sometimes referred to as intercorporeality. This implies, to offer a critical shift from dominant discourses on individualizing female lactating bodies and, instead to frame breastfeeding and milk sharing as a transformative, intersubjective, and a mutually constitutive social practice. Close to the Deleuzo-Guattarian lens of *becoming-breastfeeding-woman*. towards, *becoming-breastfeeding-communities* [24].

While the mother-infant, body-to-body, bond exists in many contexts, what is particularly striking in Tzeltal communities is, there are specific intercorporeal practices such as baby-carrying for a large part of the day, where sweating, rhythms and emotional exchanges occur continuously and in mutual responsiveness. And, that this bond extends beyond the dyad, incorporating other bodies, with varying degrees of closeness, ultimately forming a kind of mother-infant-community bond.

From that perspective, the act of breastfeeding implies multiple levels of body-to-body experience, three of which we will consider:

1. **Embodied and affective dimensions of breastfeeding.** Recognizing the role of the body as both affected and affecting, as well as embodied knowledge, involves acknowledging breastfeeding as a practical skill performed between two communicating bodies. This interaction reflects forms of jointly learned practical knowledge, such as how to avoid pain or find the most comfortable and effective positions. At the same time, it highlights the relational level between mother and infant: a mutual body-to-body coupling that goes beyond nourishment to encompass a co-regulatory process. Through this closeness, both bodies co-create an affective niche that responds to nutritional, vital, behavioral, and emotional needs. Extensive evidence supports the importance of skin-to-skin contact for the well-being of both mother and child, as well as the promotion of a shared rhythmic foundation, through synchronized heart rate and parasympathetic nervous system coordination, that supports self-regulation and social engagement during early feeding practices [25, 26].
2. **Communitary dimension.** The role that bodies other than the mother or primary caregiver play, whether or not the caregiving role is shared with other members of the family or community in providing care, and whether or not they contribute to creating the conditions for the mother to breastfeed freely. The community can play a crucial role in promoting the observational learning necessary for embodied knowledge, as well as in making time and space available for women to mothering and breastfeeding.
3. **Situated contexts and spaces.** Bodies are situated in spaces and territories that have their own norms and structures. And, these spaces shape experiences through their implicit values and constraints, either encouraging or discouraging breastfeeding, formula feeding, or feelings of empowerment and shame associated. This also implies the social perception of women’s bodies in public spaces, the cultural value of this act, the construction of understanding what it means to be a mother, and the expectations of fulfilling this or other roles.

These descriptive levels help us recognize that breastfeeding bodies are complex, contested, shaped by usos y costumbres practices, and often moralized. Gaining a deeper understanding of the breastfeeding experience opens up possibilities for more effective and meaningful promotion strategies. As Hausman [19] argues, women cannot position themselves ‘on the same side’ whether they are urban, rural, or Indigenous; it becomes necessary to acknowledge differences in culture, power and privilege. The decision to breastfeed–or not–is influenced by narratives of identity, environment, social justice, and power dynamics.

## Methods

This article uses a qualitative approach and follows a systematic process, starting with an exploratory phase to lay the groundwork for further investigation. As part of a larger study that adopted a community-based, participatory approach, with the general objectives to, first, understand how lived experiences shape breastfeeding within indigenous communities (Project: “*Modelo de abordaje ecológico para la promoción del desarrollo cognitivo y corporeizado de la primera infancia IBERO 0052”*). And second, to assess embodied breastfeeding practices in San Juan Cancuc, in order to design more effective public health strategies that respect and supports local cultural practices. In this initial stage, we explore embodied breastfeeding experiences, communal practices, and barriers to breastfeeding within a Tzeltal Indigenous community in Mexico.

For the present study, we realize a cross-sectional study to document breastfeeding practices and beliefs –*usos y costumbres–* among Tzeltal mothers, emphasizing their embodied and communal dimensions. Using qualitative methods and interpretative phenomenological analysis within the framework of the Operational Research Groups, drawing on Enrique Pichon-Rivière’s classical definition: “a restricted set of people, linked by constants of time and space, articulated through mutual internal representations, oriented toward a shared task” [27]. This framework guides group interactions and reflective practices, positioning the participants as co-creators of knowledge.

In this first phase, participants collectively explored their breastfeeding experiences–embodied, relational, and communal– activating emotional, cognitive, and cultural dimensions. These processes allowed for the construction of shared meanings around maternal care within body-to-body interactions.

As Almeida [28] defines, operational groups emphasize reflection, dialogue, and transformative learning over hierarchical or extractive approaches. Knowledge arises horizontally, through interaction and mutual learning, with coordinators facilitating rather than directing the process. Allowing adaptive and responsive engagement with the group’s evolving context. From this perspective, participants do not require formal training to take part in the operative group, since its methodological strength lies precisely in the fact that, given adequate time and space to act in a relevant, cooperative, and empathetic manner, and as members of a group with a sense of belonging and willingness to learn, it becomes possible to co-construct meaningful knowledge based on the group’s lived experiences around the topic of interest.

The training required primarily concerns the group coordinator, who must possess basic listening skills and the ability to observe group dynamics in order to formulate hypotheses and questions that can be reiterated, refuted, or reformulated by the participants. In this way, the knowledge rooted in their experiences becomes explicit material for understanding reality.

Unlike traditional qualitative methods, such as focus groups, operational groups treat the group as a collective epistemic subject. This resonates with Cooperative Inquiry [29], which similarly values shared exploration and iterative cycles of reflection and action.

The practical methodological application of operational groups in this research consisted of a single intensive of four-hours session, structured around a collective task. This approach was significantly supported by the existing relationships between participants, who were neighbors and many of them also involved in a coffee cooperative, fostering pre-established bonds. These established connections, alongside with previous collaboration within the civil organization “*Educampo*”, provided a stable foundation for trust, openness, and dialogue. Interactive techniques employed during the session allowed participants to express and articulate their experiences confidently, resulting in collective meaning-making and reflection. These methods allowed capturing participants’ embodied experiences and insights within their unique socio-historical contexts, promoting understanding of the collective and individual perspectives, experiences, and actions specially those related to breastfeeding in the Tzeltal community.

By systematically integrating operational groups methodology with participatory sense-making, this research provides a comprehensive framework for investigating complex social practices. Participants actively engaged in the ongoing creation, interpretation, and transformation of knowledge, influenced and continuously reshaped by dynamic interactions within the group’s collective processes. This methodological stance tries to keep an ethical, respectful, and non-extractive engagement with communities, explicitly rejecting epistemic extractivism to mantain a collaborative inquiry that is both equitable and reciprocal.

### Participants: sociodemographic profile of the study participants

A total of 19 women participated in the operational research group conducted in San Juan Cancuc. All participants were mothers, and –except for those over 60 years of age (2)– all were currently breastfeeding at the time of the session. The participants ranged in age from 16 to 70 years old, with an average age of 41.4 years and a median age of 35. This age distribution reflects a diverse group of women. Many of the women (14) mentioned having more than two children.

All of the women are engaged in household tasks. The association that facilitated our contact encourages women to take on a greater role in other activities, including those related to coffee cultivation. All the women present belong to coffee-growing families, and some of them own their own plots. Consequently, their time is divided between household chores, helping in the fields, and, more occasionally, leaving the community to sell products.

The nearest urban center is San Cristóbal de Las Casas, where the Spanish-speaking women reported going to sell. Outside this context, the women tend to remain within their community. Participation was voluntary and with no financial compensation. All the participants gave their written informed consent. The study was approved by the Iberoamerican Research Ethics Committee (CEl:9/24).

All participants were native Tzeltal speakers, and only two of them were also fluent in Spanish, highlighting the linguistic and cultural specificity of the group. While formal education levels were not recorded, the group dynamics and testimonies underscored a strong base of experiential knowledge shared among the participants.

Due to the cultural characteristics the session was entirely in Tzeltal emphasizing the embedded cultural, oral, and visual forms of dialogue. The narratives that appear in the third person are due to the fact that the responses were given in the Tzeltal language; where it is common to speak in the third person even when referring to a first-person experience.

The shared experience of motherhood and breastfeeding provided a unifying framework for the conversation, fostering trust and openness throughout the session. This sociodemographic composition, rooted in Indigenous language, motherhood, and community roles, deeply shaped the content and structure of the research process.

The working group included an on-site translator; a man who acted as both technician and community liaison for Educampo’s coffee production projects in the region, and a native Tzeltal speaker who had participated in many previous initiatives and agreed to support the intervention. The mediator was someone with already established relationships in the community. The coordination of the operational team was led by one of the researchers, who had extensive methodological expertise, having been trained at the El Tigre School of Social Psychology founded by Alfredo Moffatt in Argentina. He also had experience in intercultural contexts, working at a university in Chiapas and previously engaging in projects within the region. In addition to them, the representative from Educampo was also present, with the purpose of strengthening the relationship between the coordinator and the community.

### Operational research group

The operational research group was conducted on November 12, 2024, in the Tzeltal community of San Juan Cancuc. The session was structured as a participatory and emergent dialogue, grounded in the Operational Group methodology, which posits that each group naturally develops internal leadership and forms spokespersons for collective meaning-making. Throughout the session, non-verbal cues, such as smiles, nods, and body language, were observed as indicators of agreement, signaling the affective and collective dimensions of breastfeeding as a shared experience.

Most women breastfed at different moments during the meeting, allowing for direct participant observation of breastfeeding as a communal and embodied practice rather than an isolated maternal act.

The session structure consisted of two core components:

1. The collective construction of breastfeeding embodied meanings through participatory dialogue.
2. A consultation with a IBCLC (international Board Certified Lactation Consultant), responding to emerging concerns raised by participants.

The consultation with the IBCLC was based on the hypothesis that it could serve as a viable form of social retribution for the women in this community, to avoid epistemic extractivism. However, for reasons that will be discussed later, this component turned out to be of little relevance, as most of its proposals –such as milk expression or addressing breastfeeding impediments – were not suitable for the local context.

### Sensory memory exercise and guided meditation

As part of the participatory research approach, the session began with a sensory memory exercise and guided meditation designed to evoke early-life memories related to maternal bonding, physical affection, and embodied memory. This exercise was not only a methodological tool for introspection but also a catalyst for embodied engagement with the breastfeeding dialogue, within affective and intergenerational experiences.

The meditation exercise prompted participants to recall and reflect on:

- The first time when they saw their mother, engaging both visual memory and multisensory recall (smells, sounds, and skin sensations).
- Their first interactions with other family and community members, and the moment they first heard their own name.
- Recognizing faces, flavors, and sounds from childhood, evoking early sensory imprints.
- The first time they were hugged and the first time they witnessed joy in other´s face.
- The first time they embraced their own child, recalling the emotional charge of maternal initiation.
- How their bodies experienced transformation during the initial stages of breastfeeding.

Although the meditation was initially proposed as a silent exercise, participants spontaneously engaged in conversation, building together breastfeeding meanings even before the formal discussion began. This unstructured interaction aligned with the principles of Operational Groups, where some members manifest bodily expressions (e.g., breastfeeding during the session), while others verbalize collective experiences through dialogue.

Following the meditation, each participant was invited to articulate a single word that encapsulated the emotions and memories evoked. This word served as an entry point for the subsequent discussion on breastfeeding, bodily experiences, and maternal identity.

## Data collection and analysis

The session was documented through field notes and audio recordings, allowing for qualitative content analysis. We applied a conventional content analysis with emergent coding, following [30], which enabled us to inductively generate categories directly from participants’ narratives, ensuring that the analysis remained grounded in their lived experiences and points of view, rather than imposing predefined or external structures.

Field notes were taken by the group coordinator and later cross-checked with the translator, the Educampo representative, and the IBCLC colleague to identify possible errors or omissions and ensure a faithful account of the process and its followed the guidelines established by the operational research group methodology and focused on systematically observing group phenomena from a situated perspective, acknowledging that all observation is shaped by the observer’s subjectivity, which is understood as an operative tool rather than a bias to be eliminated. Communicational exchanges were documented in both verbal and nonverbal dimensions–dialogues, silences, gestures, tone of voice, laughter, or tension – along with the affective climates, presences and absences, and the group’s reactions to them.

This approach maintained methodological fidelity to spontaneous, participatory, and emergent qualitative inquiry, allowing for a deeper exploration of the affective, bodily, and social dimensions of breastfeeding as experienced and expressed by the participants themselves.

### Thematic inquiry, codification and emergent discussion

The discussion that followed was not pre-scripted; rather, the guiding questions emerged organically from the participants’ own narratives and interactions and open coding was followed. The facilitator encouraged exploration of naturally occurring topics, fostering an iterative dialogue where each contribution informed the next (See Table 1).

As the conversation unfolded, recurring topics became apparent and the facilitator retrospectively identified key thematic axes that shaped the dialogue. These topics were later organized into interpretative categories:

**Table 1:** Interpretative categories, guiding questions, and operational definitions (authors’ own elaboration)

Interpretative Category	Guiding Question(s)	Operational Definition
Embodied and affective dimensions of breastfeeding	− How did your body feel the first time you breastfed - What physical sensations do you experience while breastfeeding? - How did you learn to breastfeed? - How do fatigue and daily activities interact with breastfeeding?	Bodily and emotional experiences emerging from the skin-to-skin contact between mother and infant during breastfeeding.
Memories and emotions of motherhood	- What are you feeling now with your child in your arms? - If you had to choose one word to represent what you remember, what would it be? - What does that word symbolize for you?	Emotional memories and meanings evoked while holding, caring for, and bonding with the child during breastfeeding.
Emotional and relational aspects of breastfeeding	- What does joy mean to you in relation to breastfeeding? - How does it feel to see your child looking at you while breastfeeding?	Emotional and relational meanings constructed through mutual gaze and interaction during breastfeeding moments.
Mother-child relationship and the meaning of breastfeeding	- How do you feel while breastfeeding? - What does breastfeeding mean to your relationship with your child? - What does breastfeeding mean to your relationship with your mother or other relatives?	Reflections on the significance of breastfeeding in building and sustaining the mother-child relationship, and the intergenerational one.
Customs, beliefs, and cultural perceptions of breastfeeding	- Why do some mothers continue breastfeeding for extended periods? - What do you think about stopping breastfeeding when a child begins school? - Do you like this tradition? Why?	Shared traditions and beliefs that shape how breastfeeding is practiced, valued, and continued within the community.
Community support and contextual and spatial differences	- Do you feel that your family and community support your breastfeeding? - When you are at home, do you feel judged for breastfeeding, or do you feel at ease?	Perceptions of support or judgment in different social spaces, from home to public or urban settings.

## Results

The findings of this study coming from the three key bodily and emotional aspects of breastfeeding dimensions were organized into the interpretative categories, based on the responses and experiences shared by the participants. These dimensions include the bodily and emotional aspects of breastfeeding, the community dimension of breastfeeding practices, the intergenerational transmission of breastfeeding knowledge, and the situational contexts in which breastfeeding occurs. Within each dimension, specific themes were identified through the analysis of both direct testimonies and observations, and then grouped highlighting common patterns and significant insights regarding maternal-child interactions and societal influences on breastfeeding practices.

Our results support the following: (1) Mothers within a strong community support network reported fully complying with UNICEF’s recommendation to breastfeed up to two years, with some continuing for as long as three years. This contrasts with the fact that only 31% of children under six months of age receive exclusive breastfeeding [30]. And (2) community contexts facilitate body-to-body interactions, more ways to share affection when enabling on-demand breastfeeding and collective infant care more effectively than urban settings. In contrast, urban environments, often shaped by disapproving gazes and moralized perceptions, tend to hinder breastfeeding practices [31].

### Embodied and affective dimension

Participants described various embodied physical sensations related to breastfeeding. During the initial stages, many reported experiencing tingling and, in some cases, discomfort, especially when their babies began to develop teeth. They also mentioned fatigue and drowsiness after prolonged breastfeeding sessions, alternating it with other daily activities such as washing clothes, bathing, or preparing tortillas.

Regarding the baby’s weight, some mothers explained that when they felt physically tired from carrying their children, they would lay them down in a safe place before resuming other tasks. Breastfeeding occurred at various moments without a fixed schedule, directly responding to the baby’s signals, whether through gestures or crying.

One of the most frequent expressions was that women breastfeed because they felt sorry for their crying babies. During a visual recording exercise, one participant wrote on behalf of the group, highlighting the emotion of happiness felt through her body, addressing embodied and affective dimensions. Women observed that while breastfeeding, they felt joy seeing their babies smile and the sense of tranquility that close physical contact generated, but also fatigue, which they linked more to drowsiness than to physical exertion. Conversations listened by the translator revealed that many women were concerned about their own nutrition and how it might affect their breast milk.

One participant expressed:

For babies to be nourished so they can grow, so they don’t go hungry, and so they don’t throw tantrums. When women are working, harvesting, and their baby starts crying, they stop working and breastfeed them, even if it is late.

This testimony reflects how breastfeeding adapts to the rhythm of work and other responsibilities, without a strict separation between the act of breastfeeding and the rest of daily activities.

Another participant shared her experience about their body-to-body connection between breastfeeding, the baby’s hunger, and maternal response:

Well, the baby cries, and the mother hugs them because she feels sorry or compasion for them. She feels that they are hungry and that they still can’t talk or express what they feel, so she hugs them and breastfeeds them. So the milk reaches their stomach. And that makes the mother happy when she sees her baby drinking milk and when they are well. They also feel happy when they see their children calm.

This testimony reinforces that breastfeeding is not only an act of feeding but is closely linked to the comfort and tranquility of both the baby and the mother. The immediate response to crying through breastfeeding appears to be an affective and bodily practice, where physical contact and observing the baby’s well-being generate satisfaction for the mothers.

### Communitary dimension. Community and intergenerational transmission dimension

As noted in the background and supported by our findings, participants consistently perceive breastfeeding as a primary responsibility-one that takes precedence over other work-related activities. They describe interrupting their tasks to breastfeed their babies when they cry, illustrating how maternal care is interwoven with their daily routines, and how it takes precedence in their attention. As one woman explained: “The women who are working—when the baby starts crying, they leave their work to breastfeed, and then the baby calms down and stops crying.”

On the one hand, participants explained that they learned to breastfeed by observing their mothers and grandmothers do so in public spaces, without shame or hesitation. In their community, it is common to see women breastfeeding openly, making the practice both visible and naturally transmitted through everyday life. As a form of embodied and communal knowledge that is learned by observing.

On the other hand, the increasing integration of breastfeeding women into the labor market –a behavior and set of customs that will require deeper exploration in the later phases of this project – is also connected to the economic structures shaping these communities. This shift intersects with the widespread use of complementary feeding practices and the easy or difficult access to high protein foods.

Participants reported that they maintain exclusive breastfeeding for the first six months. After this period, they begin introducing *pozol,* a traditional liquid made from corn dough, sometimes mixed with cacao and occasionally fermented. From the end of the first year, children begin consuming fruits and small portions of meat. Breastfeeding often continues up to three years, depending on the number of children. Women prioritize breastfeeding the youngest child, which means that the amount of milk each child receives depends on their birth order. However, participants emphasized that breastfeeding is always sustained throughout the first year.

### Spaces and situated context

When asked if they had ever felt judged or stigmatized for breastfeeding in public, many women reacted with surprise at the question, not recalling situations where breastfeeding had been questioned. In their community, breastfeeding is a common and visible practice in various spaces.

One participant commented that when traveling to San Cristóbal de las Casas, the nearest city, she had noticed different reactions when breastfeeding in public. However, she said this did not affect her decision to continue breastfeeding openly.

During the session, one of the women stated:

All the women here are used to breastfeeding our children, and we are not ashamed. That means that wherever we are, we can breastfeed our children, and we are not ashamed to take them out. We are used to it.

This testimony illustrates the normalization of breastfeeding within the community and the absence of inhibitions regarding the practice in shared spaces.

### Breastfeeding context and its association with economic limitations

During the session, one of the women shared her view on the differences in breastfeeding practices between rural and urban communities. She reflected on how, in cities, women may have access to other feeding options for their children, whereas in rural areas, those options are more limited.

Well, there’s a woman who doesn’t have children anymore, but she has a lot of experience to talk about. She sees that there is no difference among women in the communities or in the cities. After all, they can give their milk or give something else for the baby to sleep, because they have a little money. But in the communities, we don’t have money to buy things, so we have to sacrifice and find ways to nourish our children. We have to work. There is a difference between the city and us.

When probing the meaning of “nourish,” it was confirmed –through nods and affirmative gestures– that the women were referring to commercial milk formula. What can be inferred from this statement is that, although breastfeeding is maintained as a tradition, some women are not fully convinced that it is the healthiest option for their children and would transition to infant formula if they had the economic means to do so.

#### Coordinator observations

The aspects described above are grounded in literal transcriptions extracted from group session recordings. However, as part of the operational group methodology, there are group hypotheses that are either confirmed or rejected, as well as relevant elements that were not captured by the audio recorder –either due to microphone limitations or because they emerged outside the formal session. Aware that these operate on a different level of validation and analysis, we present them here as relevant field notes from the group coordinator, though not on the same evidentiary level as direct participant testimony.

The first observation relates to the normalization of public breastfeeding, which was visibly confirmed while walking the streets of San Juan Cancuc. The coordinator, a researcher familiar with various regions of Chiapas, has also witnessed similar practices in public spaces of larger cities like Tuxtla Gutiérrez and San Cristóbal de las Casas, including marketplaces. These everyday scenes align with the testimonies of the women who participated in the session.

A second element that did not clearly appear in the transcripts but was overheard during informal movement among the participants is the perception of a division of responsibilities: while women focus on childcare, men often assume complementary tasks to enable breastfeeding. This theme was not deeply explored in the group itself, which concentrated primarily on the mother–child–community dynamic, but it suggests a potential line of inquiry regarding gendered complementarity as a cultural support for breastfeeding.

The final conversation with the breastfeeding consultant, which occurred after the recorded group session, is not included as part of the core research instrument. However, in subsequent informal conversations, a recurring issue emerged: while the women’s breastfeeding practices align closely with many public health policy recommendations, it remains unclear whether they value these practices as optimal or see them as outcomes of economic marginalization, as noted in the section on breastfeeding and economic limitations. A working hypothesis for our future research is whether one challenge for indigenous communities is affirming the value of their traditional practices in the face of developmentalist models of parenting, in a global context, where the use of commercial milk formulas as a means of feeding young children is normalized due to the extensive marketing of these products –marketing that is increasingly diverse, sophisticated, and resourceful– this has a significant influence on beliefs, values, and practices.

During a shared meal after the session, the coordinator observed several women drinking soda, highlighting a broader concern in rural Chiapas about rising soda consumption in Indigenous territories. Chiapas, particularly San Juan Chamula, another Tzeltal-Tzotzil community, has the world’s highest per capita Coca-Cola consumption, averaging 821.25 liters per person annually. Informants suggest soda is replacing *pozol,* a traditional corn-based drink, due to its convenience. Although not directly discussed in the session, this observation raises questions about how industrialized food products may displace traditional nourishment practices and whether public or international policies that increase household income might unintentionally undermine breastfeeding and complementary feeding in favor of commercial alternatives.

Lastly, the coordinator made a visual and embodied observation that complements the verbal testimonies: many women had more than one child present during the session. These children moved freely throughout the room, climbed onto their mothers’ laps, asked to breastfeed, and played openly. There was no sign of inhibition in exposing the breast—even in the presence of two men (the coordinator and the translator). These nonverbal dynamics support participants’ claims that breastfeeding is not stigmatized or hidden, but rather normalized and seamlessly integrated into everyday life.

## Discussion

The study explores the embodied breastfeeding experiences of Tzeltal women in the San Juan Cancuc indigenous community in Chiapas. For Tzeltal women, it made visible the opportunities for these women to become more aware of and articulate their embodied breastfeeding experiences and knowledge. For health professionals, this approach encourages to identify elements that could positively impact the mother-infant relationship. And, it advocates for better strategies to promote breastfeeding practices in indigenous contexts, emphasizing the use of culturally relevant language, cosmogonic and communitarian understandings.

Through the mothers’ narratives in this study, the embodied experience of breastfeeding became evident along the proposed dimensions. As other authors have noted, focusing research on maternal subjective experience deepens our understanding of women’s choices regarding breastfeeding [32]. Such perspectives can help address concerns about breastfeeding initiation and duration, including factors such as the embodied desire (or lack thereof) for skin-to-skin closeness. Moreover, they can inform the development of more effective and culturally sensitive social policies.

First, when participants describe embodied sensations –such as satisfaction, tingling, discomfort, fatigue, or the physical strain of carrying their children – they highlight the need to focus research on the physical and affective dimensions of breastfeeding, rooted in the mother’s body and her embodied bond with the infant. Among Tzeltal women, this involves making sense of the affective and lived experiences of breastfeeding, including feelings of happiness, compassion, sorrow, or tranquility, as well as emotional responses that resemble empathy –expressed through their concern and commitment to the well-being of another vulnerable living body through enabling on-demand breastfeeding, for instance. Several women emphasize the importance of listening to the infant’s needs, even above agricultural work. The literature supports this kinds of body-to-body communication, that such attentive responsiveness to the infant’s signals is associated with greater emotional self-regulation in babies, a stronger affective bond within the dyad, and longer breastfeeding duration [25, 26].

These findings parallel long-standing Western beliefs surrounding breastfeeding practices since the late eighteenth century, when advice manuals emphasized maternal pleasure and the affective bonds of motherhood [31]. Likewise, with researchers such as [33] illustrate that, within the Yoruba belief system, the breast is symbolically understood not only as a vital source of nourishment but also as a medium for nurturing both the corporeal and spiritual bond between mother and child. They also align with physiological perspectives underlining the hormonal and psychological benefits of breastfeeding in this inter-affectivity niche, including the release of prolactin and oxytocin –hormones with antidepressant and anxiolytic properties that help regulate maternal stress by lowering cortisol levels, particularly when combined with skin-to-skin contact [34].

Second, recognizing embodied knowledge among Tzeltal women reveals that decisions to initiate or continue breastfeeding appear to be influenced more by intergenerational embodied learning, acquired through observing mothers and other women, than by formal advice or instruction. As [35] suggest, the knowledge, confidence, and commitment required for breastfeeding may be more effectively developed through an antenatal apprenticeship with a breastfeeding mother than through IBCLC or written materials. Among Tzeltal women, breastfeeding as a bodily ability is learned and cultivated by observing how others carry it out within the specific conditions and practices of their community.

Third, the multiple levels of body-to-body experience described through the voices of Tzeltal women also relate to shared experiences that may be deeply connected –blurring the boundaries between self and other – or, at times, painful, distressing, and disruptive [36]. Moreover, we observed that intercorporeal, or body-to-body, socialization emerges not only from the mother–infant dyad but also from the familiarity of communal environments, which allow embodied practices to develop collectively, an observation that resonates with Bateson and Mead’s [37] study of Balinese communities.

Our results aligns with that of Zapotec anthropologist Jaime Martínez Luna [38], who defines communality as ‘an experiential concept that allows for the integral, holistic, natural, and collective understanding of life’ (p. 100). Similarly, Zapotec farmer Plutarco Aquino Zacarías [39] describes it as a term that unites people—’a way to name what we feel, live, express, and inherit from our grandparents’ (p. 91). Recognizing these communal ways of living reveals the symbolic bonds between the land that nourishes and the mother who cares, elements to take into consideration to promote effective interventions targeted by health systems that take into account cosmogonic frameworks.

As several authors have reported, mothers within a communal support structure experience better breastfeeding outcomes, including longer duration and stronger affective bonds. In this sense, the analyzed embodied practices not only cultivate what [40] describes as a “socio-centric” rather than “egocentric” caregiver-baby interaction but also emphasize the continuous bodily connection and deeply embodied familiarity with grandmothers and other community members, shaping a deeply rooted identity pattern. As previously mentioned, this is reflected linguistically through an integrative “we”. Furthermore, is also reflected in the widely held belief among the Tzeltal people, about the infant’s soul (*ch’ulel*) being fragile during the first two years of life. Perhaps this belief encourages the constant attentive listening, closeness, and constant care that Tzeltal women show towards infants, given that it is part of a cultural practice holds that the soul’s presence in the body is precarious during early development, requiring constant physical proximity to the infant from other bodies and the warmth of the household fire [41, 42].

This socio-centric perspective is also evident in the recognition that the mother is not merely a provider of nutrition; rather, breastfeeding is understood as a collaborative, life-sustaining practice where mothers, grandmothers, fathers, and children co-construct life based on principles of balance and complementary duality. In the testimonies, we can observe that there is a kind of collective infant care, without detracting from the central role of the mother. Drawing from [43], we highlight how Indigenous women’s knowledge sustains life. This does not mean that the responsibility falls solely on women, as in Tzeltal communities of Chiapas, men play an essential role by creating conditions that support breastfeeding through agricultural work and food provision. Breastfeeding is framed as a collective effort involving family and community support [6]. This perspective challenges the notion of breastfeeding as an individual responsibility, emphasizing its communal and interdependent nature, as also described by [33] in Yoruba communities, where significant others actively participate in supporting the child’s health.

Finally, these ideas lead us to the last dimension, organized according to our main findings: the role of space and situated contexts. This dimension highlights the affective environments that community-based breastfeeding can generate, spaces where mothers feel they are part of a collective, that they belong to a shared place. As observed during community outreach, shared territory is reaffirmed through the creation of communal spaces in which the mother–infant bond –particularly during breastfeeding – can unfold openly and confidently. Within these territories, community-friendly spaces are imbued with cultural meanings that support care and belonging. This stands in contrast to urban settings, which often reflect a spatial and social fragmentation of caregiving, further reinforced by the lack of maternity protection policies that incorporate gender and intercultural perspectives [44].

Some authors also note that mothers may experience discomfort when breastfeeding in public and may develop strategies such as using formula to avoid potential embarrassment [45]. Similarly, Tzeltal women describe how, when they travel to the city, they often sense whether breastfeeding is welcomed or not in a given setting. In this way, urban spaces can become barriers to breastfeeding. For instance, while daycare centers represent an important public policy achievement for urban working mothers, they also exemplify this division, breastfeeding is frequently constrained by workplace structures that fail to support shared caregiving. Unlike the rural areas studied, where women often carry their infants in shawls while performing daily activities, urban environments restrict such embodied interactions. This spatial segregation reflects a broader cultural contrast: in Tzeltal communities, breastfeeding remains embedded in the fabric of everyday life, whereas in urban contexts it is increasingly confined to designated spaces. As [46] observes, this spatial separation echoes a broader societal tendency to distance mothers, fathers, partners, and primary caregivers from their infants once they re-enter the labor market.

As [47] suggest breastfeeding is a body-to-body experience reflecting “collective voices and social asymmetry,” with spatial and contextual barriers limiting or promoting access [48, 49].

Moreover, our approach to this context led us to recognize not only spatial barriers but also how socio-economic factors influence feeding preferences. Some Tzeltal women express a desire for commercial milk formula, reflecting barriers similar to those in urban settings and aligning with marketing strategies from multinational companies like Nestlé. Unar-Munguía and collborators [7] also identified a rise in formula feeding in Indigenous communities, with younger mothers introducing complementary foods and liquids earlier and breastfeeding for shorter periods than older generations. These findings align with [50], who observed that while social norms in low-resource communities support breastfeeding, they do not fully endorse exclusive breastfeeding, as small amounts of solid food are commonly introduced within the first months.

Historically, some companies and the medical establishment that promoted formula in the early twentieth century as a solution to maternal inadequacies, reinforcing the idea that breastfeeding was linked to poverty and racial minorities while formula symbolized status in Euro-American societies [51, 52]. This narrative continues today, as we could hear in some testimonials, with marketing messages portraying women’s bodies as inadequate and formula as a convenient, status and socially acceptable alternative [53]. Cultural norms with respect to infant feeding are largely responsible for the decontextualization and product view of breastmilk.

In view of the mother’s narratives presented in this study, we were able to recognize the scope that implies that breastfeeding works as an embodied practice influenced by many factors, making it neither purely biological nor purely cultural as had been suggested [33]. As asserted, breastfeeding is shaped by sociocultural contexts, expectations, information, physiology, emotions, and family history [19].

For these reasons, it is essential to identify and understand breastfeeding within its sociocultural and cosmogonic context, and to provide effective, supportive, comprehensive, and contextualized care that respects each woman’s knowledge, experience, and life history –helping her navigate fears, challenges, and diverse affectivities throughout the breastfeeding process. Health professionals must therefore be equipped with an understanding of the main difficulties that may arise, along with appropriate strategies to address them, so they can offer meaningful support to mothers when needed.

Otherwise, as noted by [54] and [55], significant barriers persist in breastfeeding promotion and counseling within Mexico’s Primary Health Care system –particularly in Chiapas and, more specifically, in Tzeltal communities. Both studies highlight issues such as the shortage of trained professionals, ineffective communication, the frequent promotion of commercial formula, and the lack of informational materials in Indigenous languages. Addressing these gaps will be a key focus in future phases of this research.

With a perspective closer to our own [56], examined how cultural beliefs and practices shape breastfeeding behaviors among Indigenous women in Guatemala. They highlighted how giving birth at a midwife’s house, spending time in the *temascal* (traditional sauna), and early breastfeeding initiation were linked to increased breastfeeding frequency and better infant outcomes. In contrast [57], found that cesarean births are associated with delayed breastfeeding initiation, shorter breastfeeding durations, and increased formula use, as observed in a Maya agriculturalist community in 2014. Both studies, along with the present research, stress the importance of integrating local cultural beliefs into public health initiatives and underscore the need for geographically targeted and culturally competent breastfeeding education in Indigenous communities.

While generalizing or romanticizing these traditional breastfeeding practices should be approached with caution, in rural and historically marginalized areas like Indigenous communities in Mexico, prolonged breastfeeding is not only a cultural practice rich in body-to-body interactions but also the most economical, hygienic, and nutritionally adequate option. This is precisely why, in our research, the importance of the context, of spaces, is crucial. For example, a cross-cultural dialogue in this context could involve continuing to recognize the introduction of *pozol* after six months of age as a main complementary food, in line with national and global standards, while promoting that it is not given before that age. It also means ensuring that complementary feeding recommendations for this population includes foods that align with current recommendations –rich in high-quality protein, vitamins, and minerals – while remaining culturally acceptable and locally available.

In conjunction with such approaches, we agree that it becomes crucial to examine the lived and embodied realities, including cosmogonic, communitarian identity, and territorial structures that shape women’s breastfeeding decisions.

One of the study’s greatest strengths lies in its methodology, which enables learning that goes beyond the commonly explored perceptions within the field of public health. A potential limitation is that none of the investigators speak Tzeltal. As a result, translation was necessary, and some valuable nuances or insights may have been lost in the process. The results obtained in this qualitative study may be limited by the small sample size, which limits the generalizability of the findings to the wider population of Tzeltal communities, however despite the small sample size, we were able to demonstrate significant embodied experiences and barriers. Future studies in the other phases of this study pretend to include objective measures.

We recognize the importance of identifying barriers, but also the strengths that emerge from a deeper understanding of these communities’ idiosyncrasies. Understanding their impact on key breastfeeding-related environments offers pathways for analysis across all levels, including territories, healthcare systems, workplaces, and homes. It is essential to support Indigenous communities in preserving breastfeeding traditions while providing resources for those whose practices have been disrupted by colonialism. Indigenous groups and other marginalized communities, who have been at the forefront of reclaiming these traditions, deserve sustained support.

## Conclusions

Our study highlights the embodied and communal factors that shape breastfeeding among Tzeltal women. The findings fulfill the aim of gaining a deeper understanding of these embodied and affective breastfeeding practices, offering insights that can guide public health practitioners and community leaders in developing more context-specific policies, actions, and intercultural strategies to promote, protect, and support breastfeeding. It also contributes to the literature by exploring the social and body-to-body dynamics shaping breastfeeding practices in this context.

We realize the value of these traditional practices in the face of developmentalist models of parenting, that may be part of the current breastfeeding health promotion discourse. When it comes to understanding the obstacles to breastfeeding, women’s and babies’ bodily experiences and social relationships are often considered secondary to the primary focus on nutrition and health. However, in cultures, such as Tzeltal one, where agents experience themselves less as separate beings and more as part of a social community, quite different concepts emerge. In these contexts, breastfeeding is conceived not merely as an intra-body process but rather as a bodily, interpersonal, and communal experience.

We conclude that to improve strategies to create environments that support breastfeeding in the different contexts in the recommendations for Mexico to become a breastfeeding-friendly country, it is necessary:

- To recognize the testimonies of the embodied experiences of breastfeeding women through their embodied and situated experiential narratives.
- And, to recognize data situated in the Mexican context, such as community idiosyncrasies in parenting, specifically the role played by community care, in the Latin American understanding that it takes a whole community to care for children: along with breastfeeding narratives [58].

## Data Availability

The datasets supporting this article are included within the article and here: 10.5281/zenodo0.15468802.
